# Supplementary material for: Classically studied coherent structures only paint a partial picture of wall-bounded turbulence
Source: Nat Commun. 2025 Nov 19;16:10189. doi: 10.1038/s41467-025-65199-9 (PMC12630638; doi:10.1038/s41467-025-65199-9)
Supplement: Supplementary file 1 — Supplementary Information [file 41467_2025_65199_MOESM1_ESM.pdf]

Supplementary material for: Classically studied coherent structures only paint a partial picture of wall-bounded turbulence

Andrés Cremades[1,2*], Sergio Hoyas[1] and Ricardo Vinuesa[3,2*]

[1]Instituto Universitario de Matemática Pura y Aplicada, Universitat Politècnica de València, Valencia, 46022, Spain.
[2*]FLOW, Engineering Mechanics, KTH Royal Institute of Technology, Stockholm, SE-100 44, Sweden.
[3]Department of Aerospace Engineering, University of Michigan, Ann Arbor, MI 48109, United States.

*Corresponding author(s). E-mail(s): ancrebo@upv.es; rvinuesa@umich.edu;

Validation of the causal nature of the SHAP values

SHAP values are applied to the evolution of turbulent flows, identifying key regions that influence their development. The causal implications of the SHAP values are exploited, unveiling the cause and effect relationships inside the flow. In this section, we present a series of causal validation tests, adapted from Martínez-Sánchez et al. [1], to assess the effectiveness of the explainable deep learning methodology. Each system consists of three variables, Q_1, Q_2 and Q_3, whose values depend on their states at the previous time steps and a noise level W_1, W_2 and W_3 respectively:

$$\left.\begin{aligned} Q_1^{t+1} &= f_{Q_1}\left(Q_1^t, Q_2^t, Q_3^t\right) + g_{W_1}\left(W_1^t\right) \\ Q_2^{t+1} &= f_{Q_2}\left(Q_1^t, Q_2^t, Q_3^t\right) + g_{W_2}\left(W_2^t\right) \\ Q_3^{t+1} &= f_{Q_3}\left(Q_1^t, Q_2^t, Q_3^t\right) + g_{W_3}\left(W_3^t\right) \end{aligned}\right\} \quad (1)$$

Next, a deep-learning model, f, is trained to predict the evolution of the variables Q_1, Q_2 and Q_3: $\left[Q_1^{t+1}, Q_2^{t+1}, Q_3^{t+1}\right] = f\left(\left[Q_1^t, Q_2^t, Q_3^t\right]\right)$. The model f is a fully connected neural network with 9 hidden layers of 8 neurons each and every an output layer with 3 neurons. The model is trained on a database of 200,000 samples, with 80% used for training and 20% for testing. Finally, the SHAP values are applied to

assess the influence of the variables Q_1, Q_2 and Q_3 at time t on their evolution at time $t+1$. These values are computed over the test database and averaged to identify which input variable has the most significant impact on the predictions of the models.

The first case is a system with a mediator variable, where an intermediate variable transmits the information between the other two variables. In this system, the variable Q_1 depends on Q_2 and Q_2 depends on Q_3. The model is defined as follows:

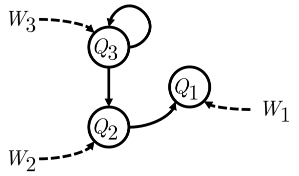

$$\left.\begin{aligned} Q_1^{t+1} &= \sin\left(Q_2^t\right) + 0.001W_1^t \\ Q_2^{t+1} &= \cos\left(Q_3^t\right) + 0.01W_2^t \\ Q_3^{t+1} &= 0.5Q_3^t + 0.1W_3^t \end{aligned}\right\} \quad (2)$$

The mean SHAP values averaged over the test data, are presented in Supplementary Figure 1. In the figure, each color represents the contribution of the input variables Q_1^t, Q_2^t and Q_3^t to the evolution of the output variables: Q_1^{t+1} (blue), Q_2^{t+1} (orange) and Q_3^{t+1} (green). The SHAP values confirm that Q_2^t is the only variable influencing Q_1^{t+1}, while Q_3^t plays the most significant role in predicting both Q_2^{t+1} and Q_3^{t+1}, as defined by equation (2).

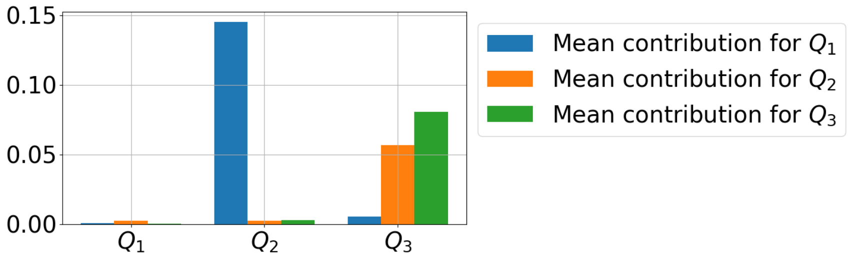

Supplementary Figure 1: **SHAP evaluation of the mediator system.** Mean SHAP value of the input variables Q_1^t, Q_2^t and Q_3^t for the model defined in equation (2), to predict their evolution Q_1^{t+1} in blue, Q_2^{t+1} in orange and Q_3^{t+1} in green.

The second case is a system with a cofounder variable. In this system, a single variable generates the other two, meaning that both variables Q_1 and Q_2 depend on Q_3. The model is defined as follows:

The results for the cofounder system, shown in Figure 2, demonstrate that the SHAP values can effectively capture the shared influence of Q_1 and Q_3 on the prediction of Q_1. They also reflect the influence of Q_2 and Q_3 on the prediction of Q_2. The predictions for Q_1 primarily depend on its own previous value, with a smaller contribution from Q_3. In contrast, the cofounder variable Q_3 has a stronger influence in the prediction of Q_2 as indicated by the orange bars. This idea can be justified by

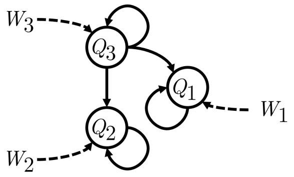

$$\left.\begin{aligned} Q_1^{t+1} &= \sin\left(Q_1^t + Q_3^t\right) + 0.001W_1^t \\ Q_2^{t+1} &= \cos\left(Q_2^t - Q_3^t\right) + 0.001W_2^t \\ Q_3^{t+1} &= 0.5Q_3^t + 0.1W_3^t \end{aligned}\right\} \tag{3}$$

analyzing the evolution of the temporal signals, Figure 3. In this figure, the strong self-dependency of the variable $Q1$ is evidenced as the high frequency of the signal Q_3 produces relatively small perturbations on the previous state of Q_1. However, the signal Q_2 presents a higher frequency which is mostly condition by the previous state of Q_3. This idea also evidences the capacity of the SHAP values not only to determine the causality between variables but also the intensity of the cause-effect relationships. Finally, Q_3 only depends on its previous value.

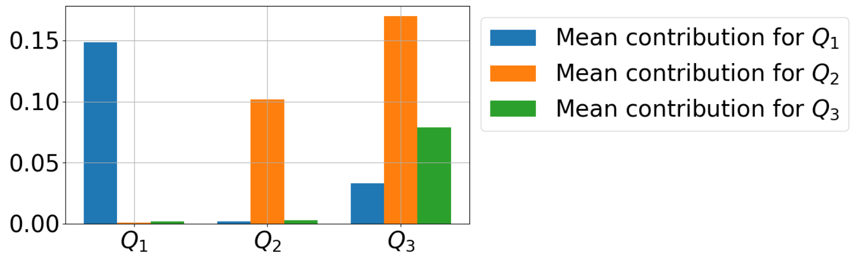

Supplementary Figure 2: **SHAP evaluation of the cofounder system.** Mean SHAP value of the input variables Q_1^t, Q_2^t and Q_3^t for the model defined in equation (3), to predict their evolution Q_1^{t+1} in blue, Q_2^{t+1} in orange and Q_3^{t+1} in green.

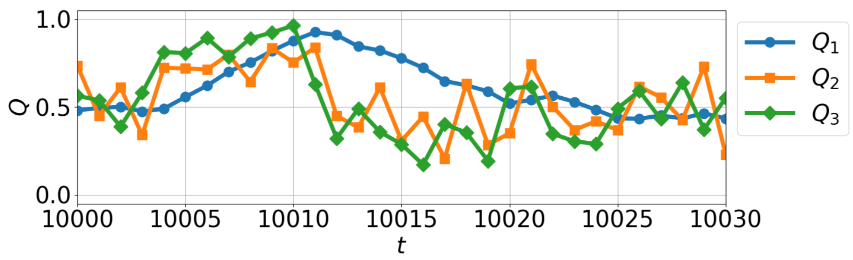

Supplementary Figure 3: **Temporal evolution of the cofounder variables.** The temporal evolution is presented for 30 consecutive time steps for the variables Q_1 in blue, Q_2 in orange and Q_3 in green. The time sampling is visualized by the position of the markers.

In the collider system, one variable depends on the other two. Specifically, the value of the variable Q_1^{t+1} is determined by the states of Q_2 and Q_3. The model is defined as follows:

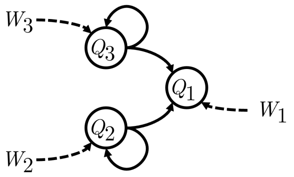

$$\left.\begin{aligned} Q_1^{t+1} &= \sin\left(Q_2^t Q_3^t\right) + 0.001 W_1^t \\ Q_2^{t+1} &= 0.5 Q_2^t + 0.1 W_2^t \\ Q_3^{t+1} &= 0.5 Q_3^t + 0.1 W_3^t \end{aligned}\right\} \quad (4)$$

The mean SHAP values presented in Figure 4 indicate that Q_1 is equally influenced by Q_2 and Q_3, while Q_2 and Q_3 depend solely on their own previous value.

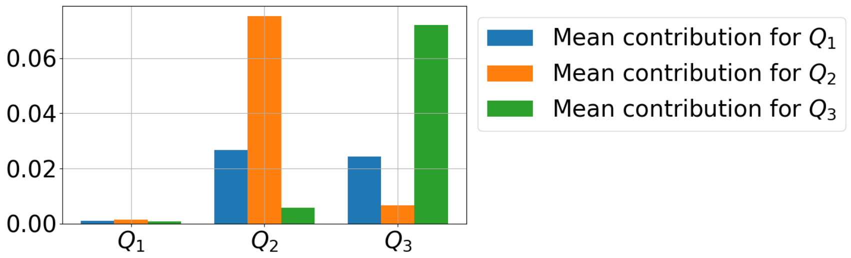

Supplementary Figure 4: SHAP evaluation of the collider system. Mean SHAP value of the input variables Q_1^t, Q_2^t and Q_3^t for the model defined in equation (4), to predict their evolution Q_1^{t+1} in blue, Q_2^{t+1} in orange and Q_3^{t+1} in green.

Finally, the redundant collider system is presented. In this case, two variables are identical, with Q_2 and Q_3 representing the same variable. The variable Q_1 depends on both Q_2 and Q_3, despite them being identical. As shown in Figure 5, the SHAP values cannot differentiate them.

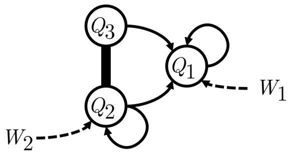

$$\left.\begin{aligned} Q_1^{t+1} &= 0.3 Q_1^t + \sin\left(Q_2^t Q_3^t\right) + 0.001 W_1^t \\ Q_2^{t+1} &= 0.5 Q_2^t + 0.1 W_2^t \\ Q_3^{t+1} &= Q_2^{t+1} \end{aligned}\right\} \quad (5)$$

Additionally, the SHAP values reveal that the influence of the sinus on the prediction of Q_1 is stronger than the effect of its previous state. The influence of the sinus in the evolution of the variable Q_1 is visualized in Figure 6, where its variation follows Q_2 and Q_3.

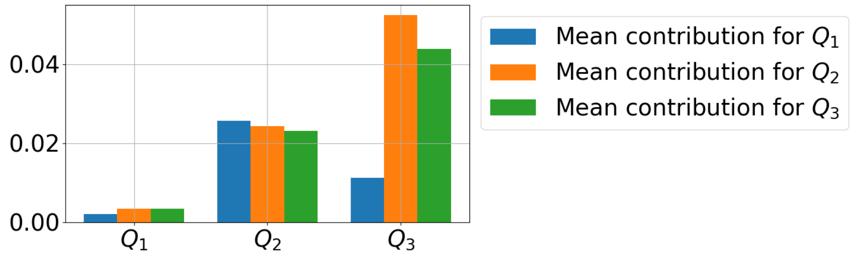

Supplementary Figure 5: **SHAP evaluation of the redundant collider system.** Mean SHAP value of the input variables Q_1^t, Q_2^t and Q_3^t for the model defined in equation (5), to predict their evolution Q_1^{t+1} in blue, Q_2^{t+1} in orange and Q_3^{t+1} in green.

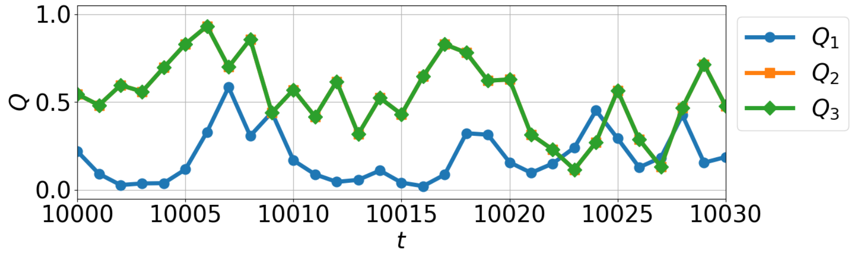

Supplementary Figure 6: **Temporal evolution of the redundant collider variables.** The temporal evolution is presented for 30 consecutive time steps for the variables Q_1 in blue, Q_2 in orange and Q_3 in green. The time sampling is visualized by the position of the markers.

SHAP values effectively capture the causal contribution of input variables in a dynamic system. Deep-learning models used to predict the evolution of a dynamic system establish causal relationships between inputs and outputs. Applying SHAP values to these models reveals these relationships by identifying the variables that have the greatest influence on the evolution of the system's state. In the turbulent channel case, grid points with higher SHAP values are those that more strongly influence the prediction of the flow's next state—in other words, they play a greater causal role in its evolution.

Noise-suppression process in the SHAP-value calculation

The high-importance regions in the flow are calculated using the gradient-SHAP algorithm, based on the expected gradients (EG) [2], which is an additive-feature-attribution method designed based on an extension of the Shapley values to a game with infinite players (Aumann-Shapley values) [3]. However, the EG generates high-frequency spatial noise, as observed in several works [2, 4, 5].

The spatial noise generated by the EG can be seen in the top-left image in the Supplementary Figure 7. This noise is a problem to generate the SHAP structures as it can break and divide them, affecting the percolation and their final identification. Therefore, to reduce this spatial noise, the periodicities of the channel in the streamwise and spanwise directions are exploited. The fields are moved along these directions, and then, the model is used to perform the same predictions subjected to the imposed displacements. A schematic representation of the model can be observed in the left box of Supplementary Figure 8. The selected periodic domain (transparent box) is translated to random positions of the original domain (light green box). Note that periodicity is exploited in this process (transparent yellow boxes).

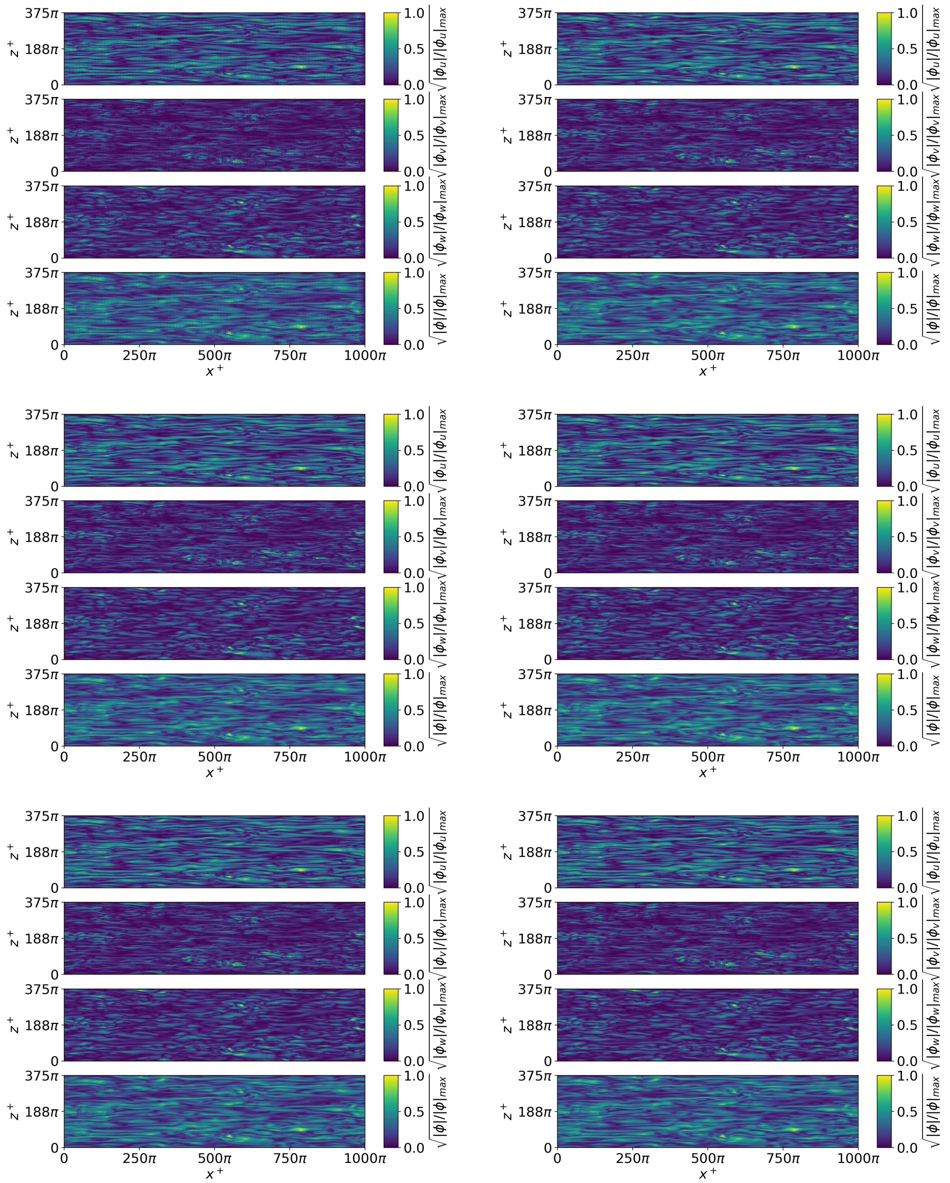

Supplementary Figure 7: **Noise reduction in the SHAP calculation.** Squared root of the instantaneous normalized SHAP-value distribution at $y^+ \approx 12$. From top to bottom, SHAP values of the streamwise ϕ_u, wall-normal ϕ_v and spanwise ϕ_w velocity components, and for the absolute value of the SHAP, $|\phi|$. Computation using single field (top-left), and with 11 (top-right), with 21 (middle-left), with 51 (middle-right), with 101 (bottom-left) and with 201 (bottom-right) translations of the field.

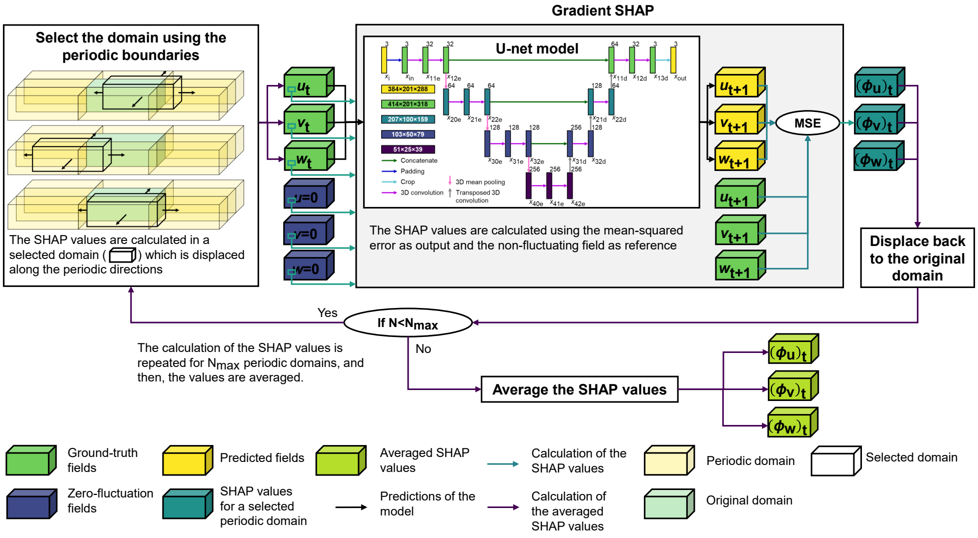

Supplementary Figure 8: **Workflow of the process to remove noise when calculating the SHAP values.** The SHAP values are calculated using the mean-squared error (MSE) of the reconstruction of the model (yellow box) over a selected periodic domain of the input field (green boxes), using a non-fluctuating field (dark blue) as reference. The flow prediction is represented with black flow connections and the SHAP-value calculation with blue flow connections. Then, the SHAP values for a selected periodic domain (blue boxes) are averaged over $N_{\max}$ periodic domains, obtaining the smoothed SHAP values (light green boxes). The selection of the periodic domain and averaging of the SHAP values are presented by the purple flow connections. The selection of the periodic domain is presented in the left box of the figure. The original domain (light green box) is replicated along the periodic boundaries (transparent yellow boxes) and a randomly centered periodic domain (transparent box) is selected for each iteration.

Then, the SHAP values are translated back to the original domain and the results are averaged. The top-right, middle, and bottom images of Figure 7 represent the improvement in the noise reduction as the number of random periodic domains is increased to 11, 21, 51, 101, and 201 translations respectively. Visually, the higher the number of translations, the better the SHAP contours are defined. However, to quantify the noise reduction, the smoothness of the solution is evaluated by calculating the sound-to-noise ratio, SNR, of each field. For this purpose, the three-dimensional fast Fourier transform of the three SHAP components, ψ_n, is calculated:

$$\psi_n\left(\mathcal{X},\mathcal{Y},\mathcal{Z}\right)=\sum_{x=0}^{N_x-1}\sum_{y=0}^{N_y-1}\sum_{z=0}^{N_z-1}\phi_n\left(x,y,z\right)e^{-i2\pi\left(\frac{x\mathcal{X}}{N_x}+\frac{y\mathcal{Y}}{N_y}+\frac{z\mathcal{Z}}{N_z}\right)},\tag{6}$$

where $\phi_n(x, y, z)$ is the original SHAP value in the spatial domain, $\psi_n(\mathcal{X}, \mathcal{Y}, \mathcal{Z})$ is the Fourier transform in the wavenumber domain and N_x, N_y, N_z are the number of samples in each direction respectively. Then, a low-pass filter is used to suppress wavenumber with a wavelength in the original domain larger than 2 consecutive grid points. The lower wavenumbers are transformed back to the original space using an inverse fast Fourier transform:

$$\phi_n^{LF}(x, y, z) = \frac{1}{N_x N_y N_z} \sum_{x=0}^{N_x-1} \sum_{y=0}^{N_y-1} \sum_{z=0}^{N_z-1} \psi_n^{LF}(\mathcal{X}, \mathcal{Y}, \mathcal{Z}) \, e^{i2\pi\left(\frac{x\mathcal{X}}{N_x} + \frac{y\mathcal{Y}}{N_y} + \frac{z\mathcal{Z}}{N_z}\right)}, \tag{7}$$

where ϕ_n^{LF} is the low-wavenumber SHAP value field and ψ_n^{LF} its Fourier transform. The noise power of the field is calculated as the difference between the power of ϕ_n and ϕ_n^{LF}:

$$\epsilon^{HF} = \phi_n^2 - \left(\phi_n^{LF}\right)^2. \tag{8}$$

Finally, the signal-to-noise ratio, SNR, is calculated as the ratio of the average value of the signal power and the noise power in decibels:

$$\mathrm{SNR} = 10 \log \frac{\overline{\left(\psi_n^{LF}\right)^2}}{\overline{\epsilon^{HF}}}. \tag{9}$$

The results of this analysis are presented in Table 1. The table shows the percentage of reconstruction of the least noisy field, obtained after 201 translations, when 1, 11, 21, 51, and 101 translataions were applied. The table evidences that a total number of 11 translations is enough to suppress the high-wavenumber noise of the solution reconstructing more than 97% of the signal for the three SHAP components, with a computational cost of approximately 5% of that of the 201 translations.

Number of translations	$\mathrm{SNR}_{u_i}/\mathrm{SNR}_{u_{201}}$	$\mathrm{SNR}_{v_i}/\mathrm{SNR}_{v_{201}}$	$\mathrm{SNR}_{w_i}/\mathrm{SNR}_{w_{201}}$
1	68.47%	60.62%	63.21%
11	98.02%	97.49%	97.22%
21	98.22%	98.30%	97.63%
51	99.40%	98.93%	99.37%
101	98.54%	98.13%	97.45%

Table 1: **Percentage of reconstruction of the signal-to-noise ratio.** The figure shows the percentage of signal-to-noise ratio reconstruction of the field with 201 translations for 1, 11, 21, 51 and 101 translations.

SHAP structures: additional information

This section shows results complementary to those presented in the main document. The definition of the SHAP structures was defined in the main paper according to a

percolation analysis which defines the structure in the regions satisfying:

$$\sqrt{\phi_u^2\left(x,y,z,t\right)+\phi_v^2\left(x,y,z,t\right)+\phi_w^2\left(x,y,z,t\right)} > H\sqrt{\overline{\phi_u^2}\left(y\right)+\overline{\phi_v^2}\left(y\right)+\overline{\phi_w^2}\left(y\right)}. \quad (10)$$

In the previous equation, the SHAP values (importance of the grid points [2, 3]) are defined by the vector $\boldsymbol{\phi} = [\phi_{\mathrm{u}}, \phi_{\mathrm{v}}, \phi_{\mathrm{w}}]$. Each component of the vector links the component of the velocity fluctuation vector $\mathbf{u} = [u, v, w]$ with its impact on the evolution of the flow. In the main text, the fluctuation of the velocity in the streamwise direction of the structures was analyzed for the whole range of wall-normal distances. The following figures show the distribution of the three velocity components for the SHAP structures (Supplementary Figure 9), the Q events or intense Reynolds stress structures [6] (Supplementary Figure 10), the streaks [7] (Supplementary Figure 11) and the vortices [8] (Supplementary Figure 13).

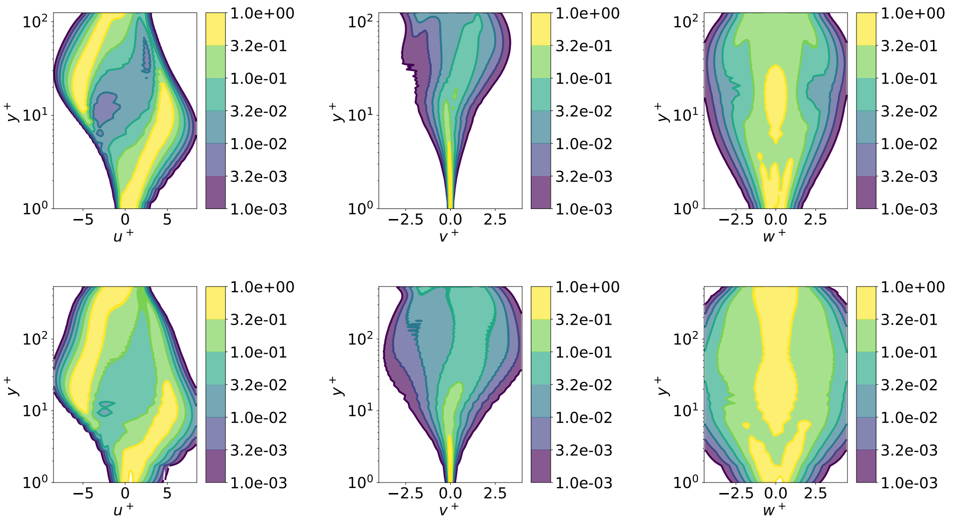

Supplementary Figure 9: **Joint probability density function of the three velocity components of the SHAP structures for the different wall-normal distances.** The figure shows the distribution of the three velocity fluctuations, namely the streamwise, wall-normal, and spanwise components from left to right for $\mathrm{Re}_\tau = 125$ (top) and $\mathrm{Re}_\tau = 550$ (bottom).

The SHAP structures exhibit a high density in the low-streamwise velocity fluctuation regions for wall-detached structures (ejection-like structures mixing with high-velocity regions) and for high-streamwise velocity fluctuation regions for wall-attached structures (sweep-like structures mixing with low-velocity regions). This idea is reinforced by the wall-normal velocity, showing the higher importance of ejections

and sweep-like structures. Furthermore, the spanwise velocities show a symmetric distribution. Most of the regions in the SHAP structures exhibit negligible wall-normal velocity fluctuations and moderate spanwise fluctuations, evidencing a strong correlation between the SHAP structures and the definition of the streaks, which are based on the streamwise and the spanwise fluctuation of the velocity. This agreement between the SHAP structures and the streaks (particularly for $y^+ \simeq 15$) was already introduced in Figure 4 of the main text. Note that the SHAP values exhibit similar distributions for both friction Reynolds numbers.

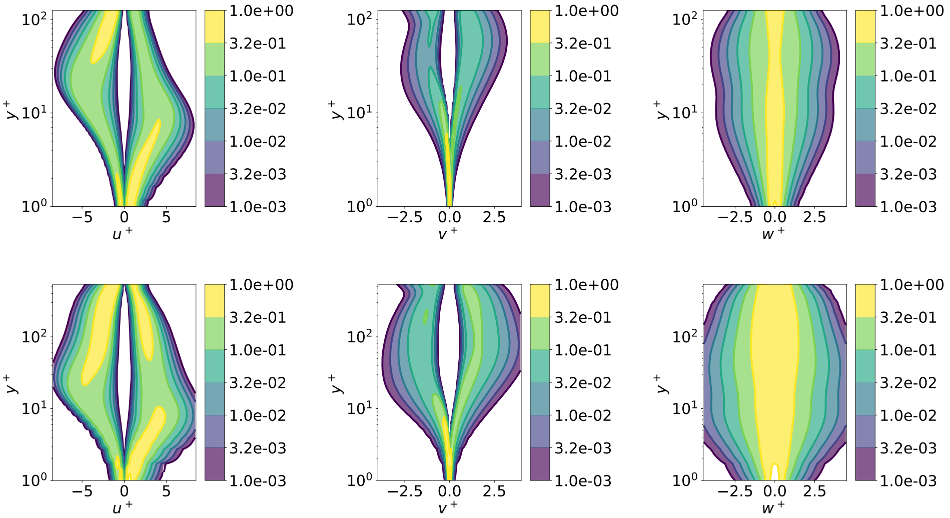

Supplementary Figure 10: **Joint probability density function of the three velocity components of the intense Reynolds stress structures for the different wall-normal distances.** The figure shows the distribution of the three velocity fluctuations, namely the streamwise, wall-normal, and spanwise components from left to right for $\mathrm{Re}_\tau = 125$ (top) and $\mathrm{Re}_\tau = 550$ (bottom).

Supplementary Figure 10 shows the velocity distribution of the intense Reynolds-stress regions. Most of these structures are located near the wall and exhibit low-velocity fluctuations. Additionally, the sweeps ($u > 0$ and $v < 0$) are found for lower wall-normal distances than the ejections ($u < 0$ and $v > 0$). This is consistent with the previously presented SHAP structures, which point to higher-importance regions where the sweeps enter the higher-local-shear stress regions (near the wall) and when the ejections mix with the lower local-shear-stress regions (far from the wall). However, although in the case of $\mathrm{Re}_\tau = 550$ a high probability of sweeps far from the wall is observed, these do not correspond to high-importance regions, which remain similar for both friction Reynolds numbers. In addition, in the main article, the SHAP structures were demonstrated to represent a constant percentage of the Q events. This information, added to the histogram of Supplementary Figure 9, evidences that the

important Reynolds-stress structures are those in which the streamwise velocity is important rather than those with a higher wall-normal velocity.

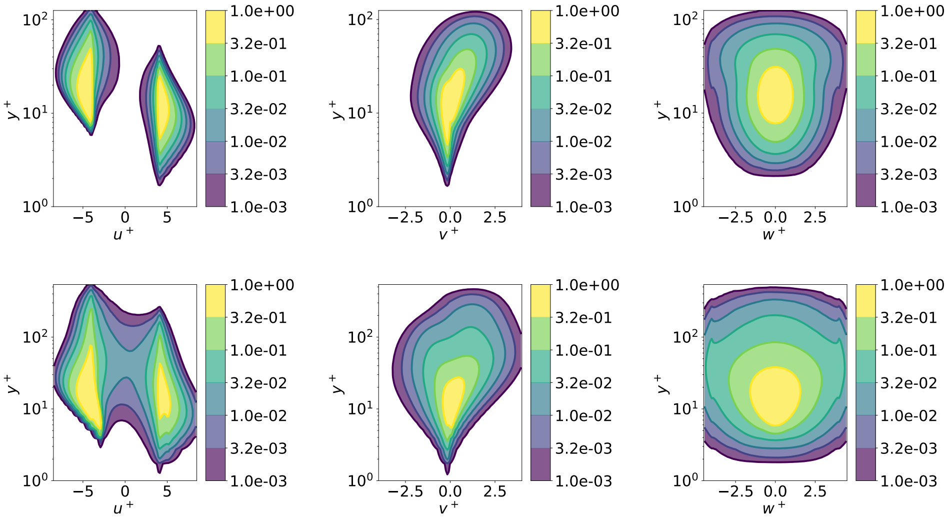

Supplementary Figure 11: **Joint probability density function of the three velocity components of the streaks for the different wall-normal distances.** The figure shows the distribution of the three velocity fluctuations, namely the streamwise, wall-normal, and spanwise components from left to right for $\mathrm{Re}_\tau = 125$ (top) and $\mathrm{Re}_\tau = 550$ (bottom).

Supplementary Figure 9 shows that the important structures are located near $y^+ \approx 15$. This wall-normal distance matches the distribution of the streaks for both friction Reynolds numbers. In addition, the distribution of the wall-normal and spanwise fluctuations is similar to that of the streaks in Supplementary Figure 11. Therefore, the SHAP structures exhibit a behavior similar to that of the ejection-like structures and sweep-like structures contained in the low- and high-velocity streaks, respectively, which have been identified by Osawa and Jiménez [9] as high-impact regions, and play an important role in turbulence transport.

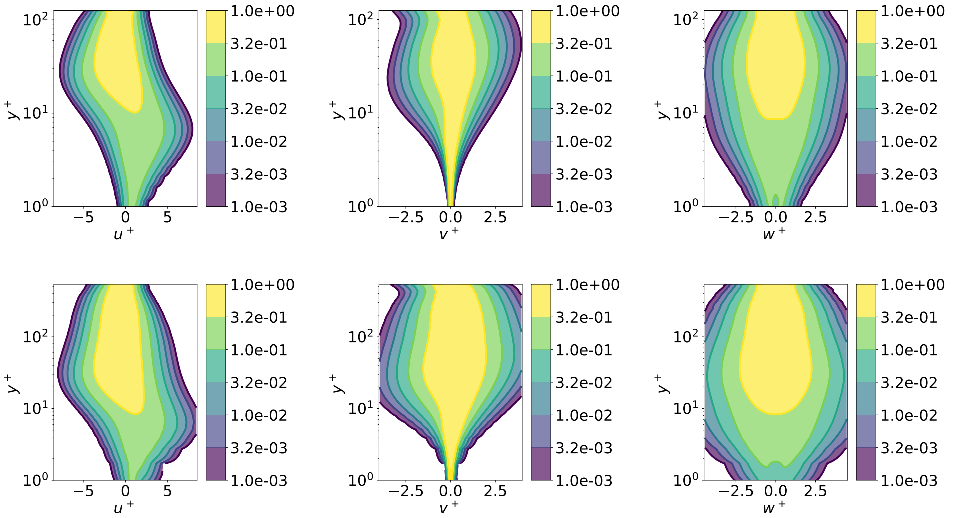

Supplementary Figure 12: **Joint probability density function of the three velocity components of the vortices for the different wall-normal distances.** The figure shows the distribution of the three velocity fluctuations, namely the streamwise, wall-normal, and spanwise components from left to right for $\mathrm{Re}_\tau = 125$ (top) and $\mathrm{Re}_\tau = 550$ (bottom).

Furthermore, the vortices shown in Supplementary Figure 13 mostly exhibit lower velocity fluctuations for the three components. This fact agrees with the region of the joint probability density function of the SHAP structures (Supplementary Figure 9), where they exhibit a non-negligible probability of structures in regions of low-velocity fluctuations for both friction Reynolds numbers. However, this correlation is not as significant as the agreement with the Q events and the streaks, as discussed in Figure 4 of the main manuscript.

Coincidence between different coherent structures

The percentage of agreement between the SHAP structures and the intense Reynolds-stress structures, streaks and vortices was presented with a visualization of the structures at three different wall-normal distances in Figure 5 from the main paper. This section focuses on extending the visualization of the structures at different wall-normal distances for the whole channel size, Supplementary Figures 13, to 17.

For a friction Reynolds number of $\mathrm{Re}_\tau = 125$, at $y^+ \approx 3$ most of the SHAP structures are composed by intense Reynolds-stress structures or Q events, with a small presence of streaks and vortices, see Supplementary Figures 13 and 14. Nevertheless, there is a large fraction of these Q events that do not match the definition of the SHAP structures. As the wall-normal distance is increased to $y^+ \approx 6$, the streaks gain importance and there is a strong agreement between the SHAP structures and

the regions in which Q events and streaks collide; note that this trend can also be observed for $y^+ \approx 35$. Then, for a wall-normal distance $y^+ \approx 13$ the SHAP structures are located inside the streaks, mostly where they match the Q events. For larger wall-normal distances, the SHAP structures are mostly located in regions of intense Reynolds stress, $y^+ \approx 81$ and $y^+ \approx 110$, and the vortices gain importance as the wall-normal distance increases.

For a friction Reynolds number of $\mathrm{Re}_\tau = 550$, the coincidence between the coherent structures for different wall-normal distances is presented in Supplementary Figures 15, 16 and 17. Near the wall, the intense Q events coincide with the SHAP structures. This visualization is consistent with the high-importance sweeps in Figure 3 of the main paper. As the wall-normal distance is increased, the Q events and the streaks collide and match the SHAP structures. Then, for a wall-normal distance $y^+ \approx 13$, the streaks increase and the SHAP structures are included within them. After this point, the streaks become weaker and the SHAP structures are located in the regions in which the Q events and streaks are coincident. For $y^+ > 100$ the presence of Q events and vortices increases, matching the SHAP structures part of the Q events, in agreement with the ideas presented on the main text: the SHAP values are in better agreement with the ejections far from the wall despite the increased presence of sweeps.

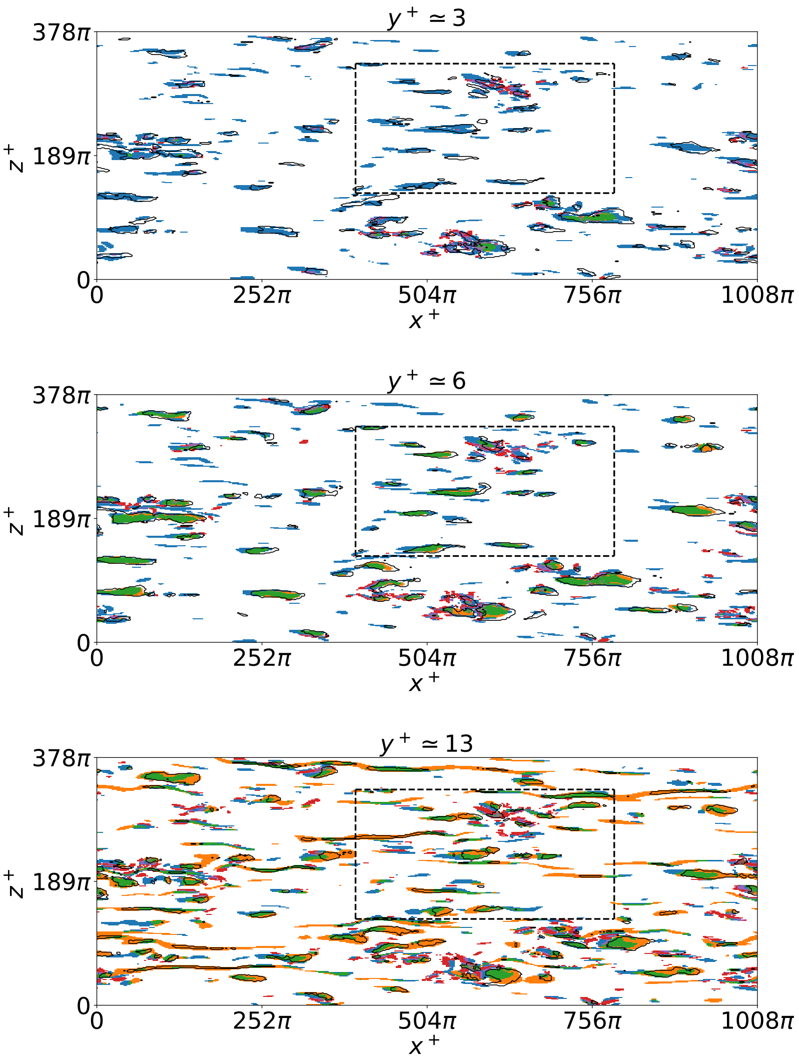

Supplementary Figure 13: **Instantaneous coincidence between Q events, streaks, and vortices for six wall-normal locations below $y^+ = 15$ for $\mathrm{Re}_\tau = 125$.** The colors used for the coincidence between structures follow this code: Qs$\backslash$(streaks ∪ vortices), streaks$\backslash$(Qs ∪ vortices), (Qs ∪ streaks)$\backslash$vortices, vortices $\backslash$(Qs ∪ streaks), (Qs ∪ vortices) $\backslash$streaks, (streaks ∪ vortices) $\backslash$Qs, Qs ∪ streaks ∪ vortices. The SHAP structures are represented by the black solid lines. Note that the dashed lines indicate the domain used in Figure 5 of the main article.

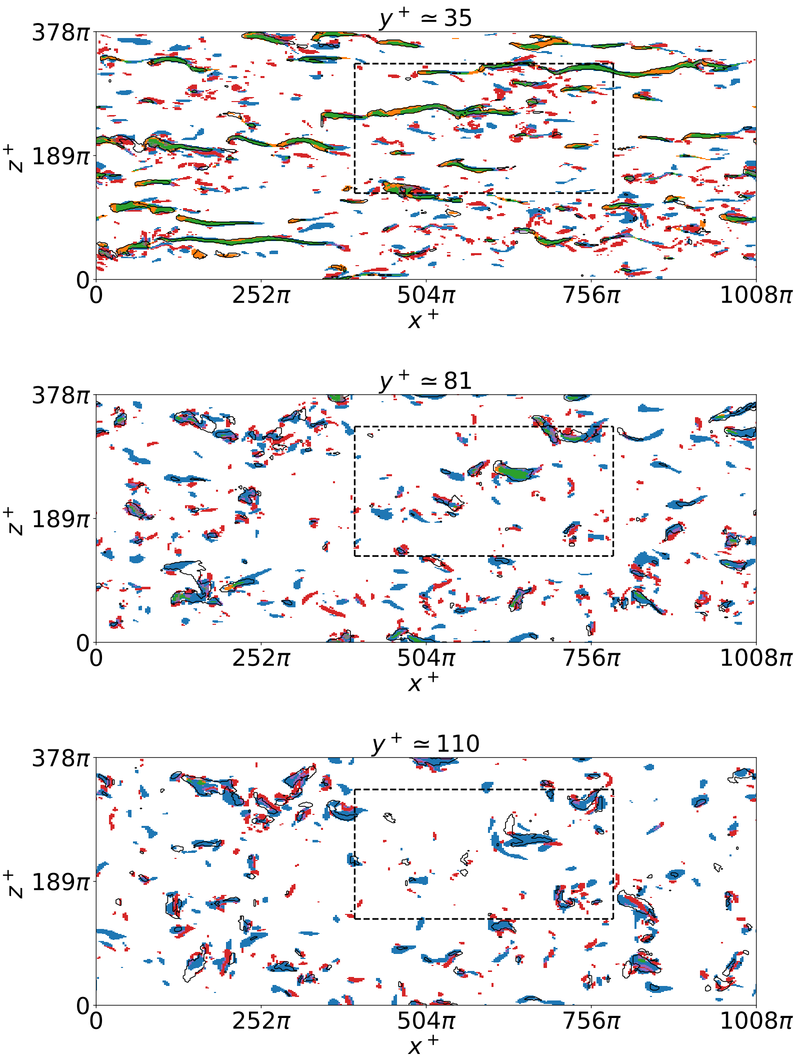

Supplementary Figure 14: Instantaneous coincidence between Q events, streaks, and vortices for three wall-normal locations above $y^+ = 15$ for $\mathrm{Re}_\tau = 125$. The colors used for the coincidence between structures follow this code: Qs$\setminus$(streaks $\cup$ vortices), streaks$\setminus$(Qs $\cup$ vortices), (Qs $\cup$ streaks)$\setminus$vortices, vortices $\setminus$(Qs $\cup$ streaks), (Qs $\cup$ vortices) $\setminus$streaks, (streaks $\cup$ vortices) $\setminus$Qs, Qs $\cup$ streaks $\cup$ vortices. The SHAP structures are represented by the black solid lines. Note that the dashed lines indicate the domain used in Figure 5 of the main article.

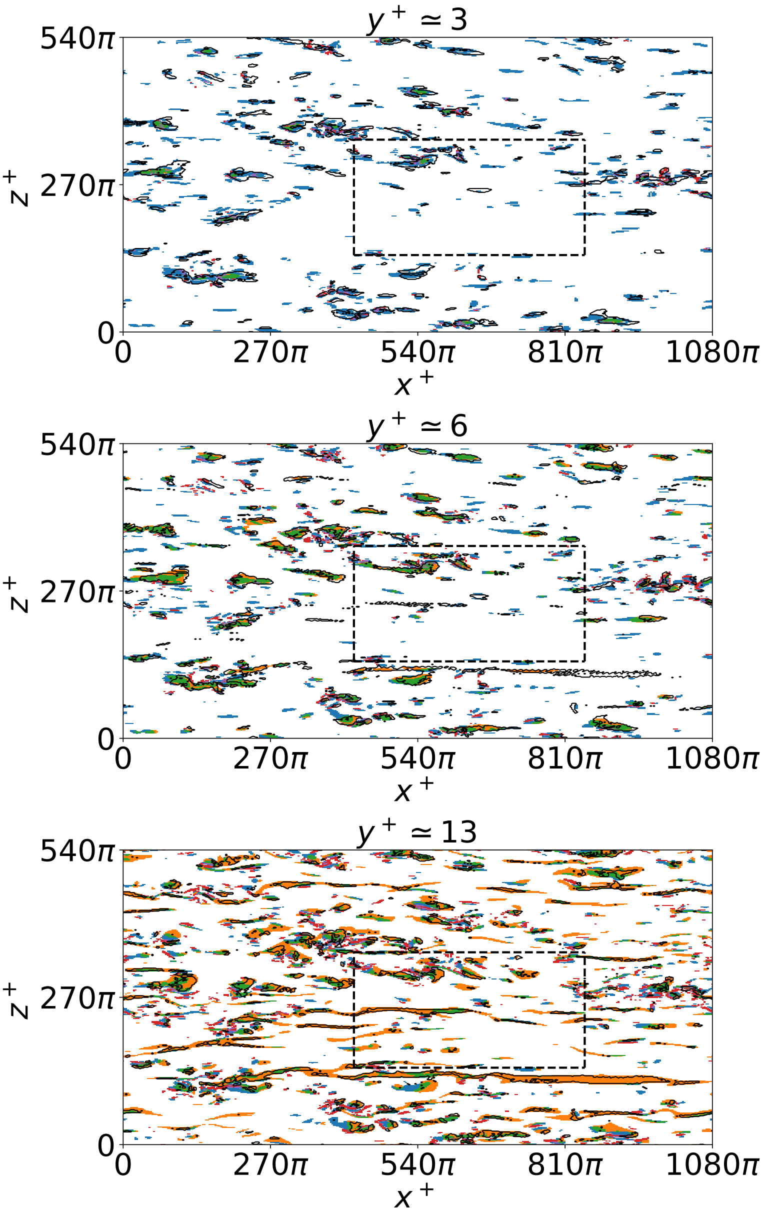

Supplementary Figure 15: **Instantaneous coincidence between Q events, streaks, and vortices for six wall-normal locations below $y^+ = 15$ for $\mathrm{Re}_\tau = 550$.** The colors used for the coincidence between structures follow this code: ■ Qs$\backslash$(streaks ∪ vortices), ■ streaks$\backslash$(Qs ∪ vortices), ■ (Qs ∪ streaks)$\backslash$vortices, ■ vortices $\backslash$(Qs ∪ streaks), ■ (Qs ∪ vortices) $\backslash$streaks, ■ (streaks ∪ vortices) $\backslash$Qs, ■ Qs ∪ streaks ∪ vortices. The SHAP structures are represented by the black solid lines. Note that the dashed lines indicate the domain used in Figure 5 of the main article.

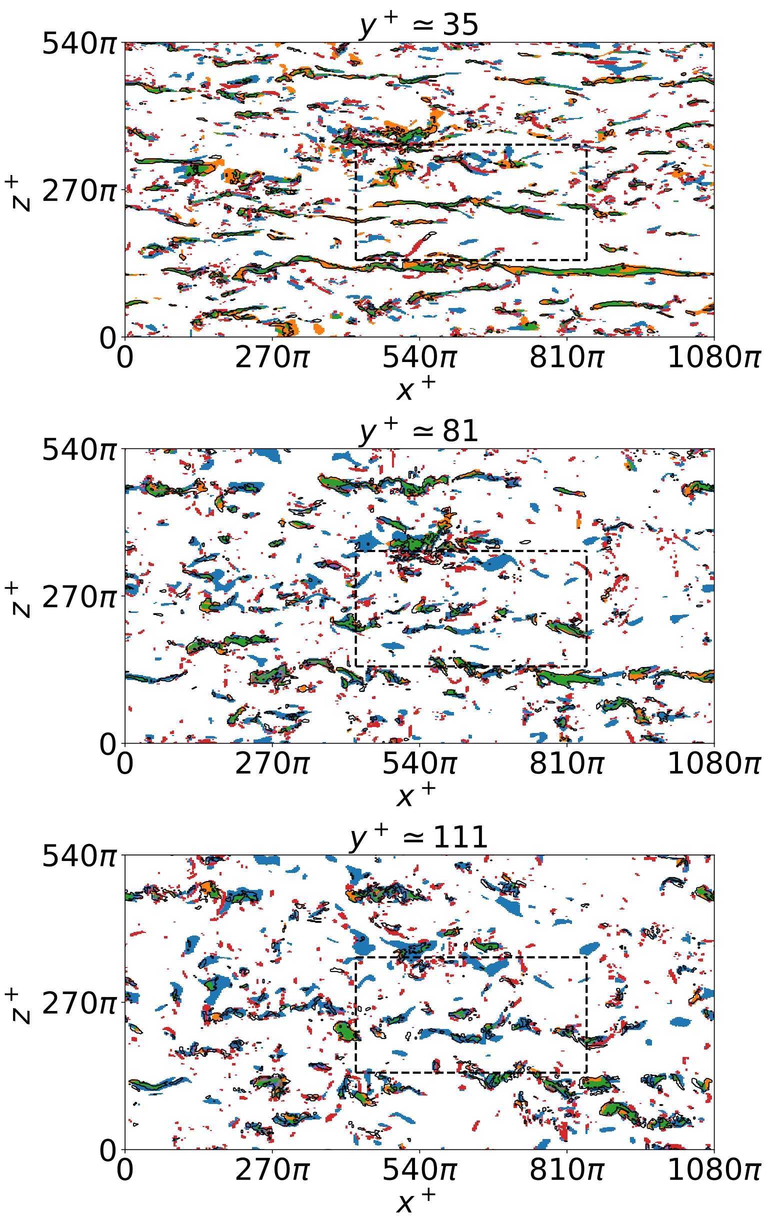

Supplementary Figure 16: **Instantaneous coincidence between Q events, streaks, and vortices for three wall-normal locations above $y^+ = 15$ for $\mathrm{Re}_\tau = 550$.** The colors used for the coincidence between structures follow this code: ■ Qs\(streaks ∪ vortices), ■ streaks\(Qs ∪ vortices), ■ (Qs ∪ streaks)\vortices, ■ vortices \(Qs ∪ streaks), ■ (Qs ∪ vortices) \streaks, ■ (streaks ∪ vortices) \Qs, ■ Qs ∪ streaks ∪ vortices. The SHAP structures are represented by the black solid lines. Note that the dashed lines indicate the domain used in Figure 5 of the main article.

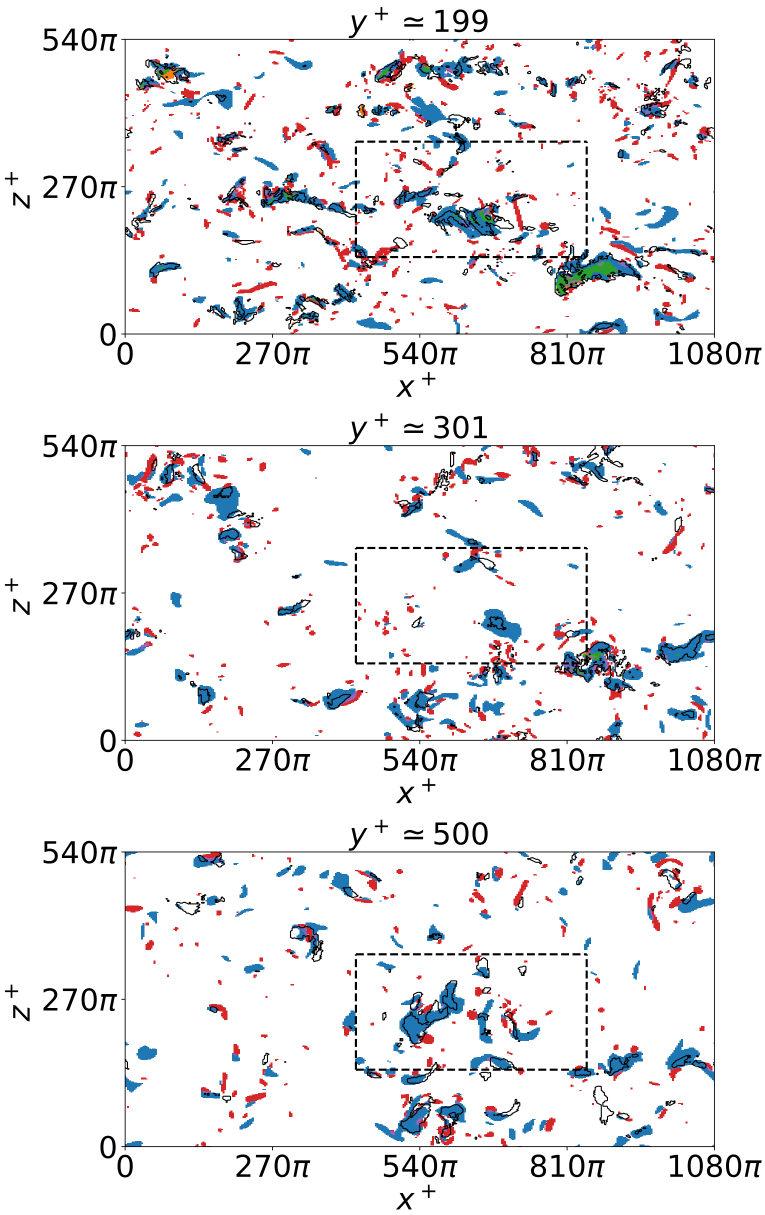

Supplementary Figure 17: **Instantaneous coincidence between Q events, streaks, and vortices for three wall-normal locations above $y^+ = 15$ for $\mathrm{Re}_\tau = 550$.** The colors used for the coincidence between structures follow this code: ■ Qs\(streaks ∪ vortices), ■ streaks\(Qs ∪ vortices), ■ (Qs ∪ streaks)\vortices, ■ vortices \(Qs ∪ streaks), ■ (Qs ∪ vortices) \streaks, ■ (streaks ∪ vortices) \Qs, ■ Qs ∪ streaks ∪ vortices. The SHAP structures are represented by the black solid lines. Note that the dashed lines indicate the domain used in Figure 5 of the main article.

SHAP structures for longer time horizons

In this section, SHAP structures are analyzed at $Re_\tau = 125$ for a longer time interval between input and output: $\Delta t^+ = 10$. The corresponding joint probability density functions are shown in Supplementary Figure 18. These distributions for $\Delta t^+ = 10$ exhibit a strong similarity with those obtained for $\Delta t^+ = 5$, as illustrated in Supplementary Figure 9. As with the shorter time step, the SHAP analysis at $\Delta t^+ = 10$ identifies regions near the wall with high velocity, and regions farther from the wall with low velocity, as most influential. Notably, for $\Delta t^+ = 10$, high-importance ejections are absent from the channel center, indicating that increasing the time interval shifts the importance toward regions with maximal velocity fluctuations, specifically around $y^+ \approx 15$.

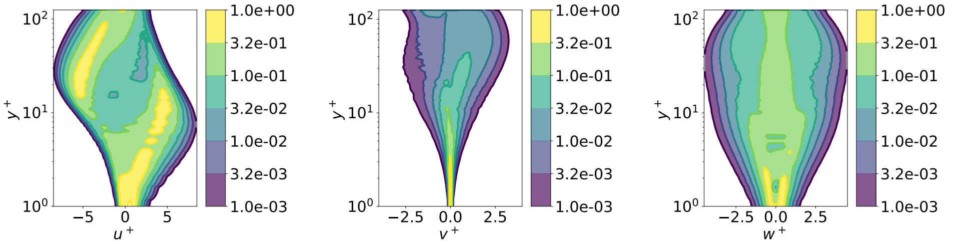

Supplementary Figure 18: **Joint probability density function of the three velocity components of the SHAP structures for the different wall-normal distances for a time horizon $\Delta t^+ = 10$.** The figure shows the distribution of the three velocity fluctuations, namely the streamwise, wall-normal, and spanwise components from left to right for $\mathrm{Re}_\tau = 125$.

Regarding the overlap between SHAP structures and traditional coherent structures, the results are very similar to the ones obtained for $\Delta t^+ = 5$, with a small reduction in agreement, particularly with the streaks, which account for a smaller proportion of the SHAP values. This observation reinforces the interpretation that SHAP values isolate the most influential regions of the flow rather than simply reproducing known structures.

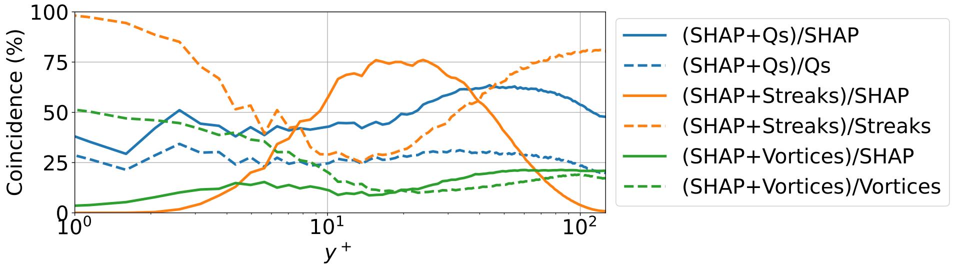

Supplementary Figure 19: **Coincidence of the coherent structures for $\Delta t^+ = 10$.** Percentage of coincidence of pairs of the following structures: SHAP, Q events, streaks and vortices, relative to the volume of each type of the pair for a turbulent channel at $Re_\tau = 125$.

SHAP structures for the evolution of the vorticity

The present analysis focuses on the evolution of vorticity in a turbulent channel flow at $Re_\tau = 125$. Here, vorticity fluctuations are temporally evolved using a U-net model, following the approach shown in Figure 1 of the main paper. SHAP values are then employed to quantify the importance of each grid point in the predictions of the model. Supplementary Figure 20 presents the normalized joint probability density function (PDF) of the velocity fluctuation components and wall-normal distance within the SHAP-identified structures. These joint PDFs reveal that vorticity transport is primarily concentrated in ejection-like structures near the wall and sweep-like structures farther from the wall. The former are associated with vorticity generation due to wall friction and lift-off effects, while the latter contribute through the downward rotational motion of the flow toward the wall.

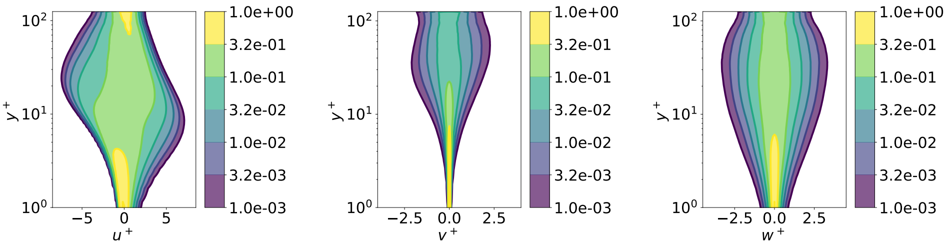

Supplementary Figure 20: **Joint probability density function of the three velocity components of the SHAP structures for the evolution of the vorticity as a function of the wall-normal distance.** The figure shows the distribution of the three velocity fluctuations, namely the streamwise, wall-normal, and spanwise components from left to right for $Re_\tau = 125$.

To investigate the instantaneous overlap between SHAP structures associated with vorticity and other coherent structures, a spatio-temporal coincidence analysis is performed. Supplementary Figure 21 shows the volumetric overlap between the SHAP-identified vorticity structures and the other flow structures, including Q events, streaks, vortices, and SHAP structures for velocity. The SHAP structures for vorticity display a low level of agreement, with less than 20% volumetric coincidence with the other coherent structures. This result highlights the power of the explainable-deep-learning framework to identify different types of relevant phenomena in high-dimensional chaotic systems.

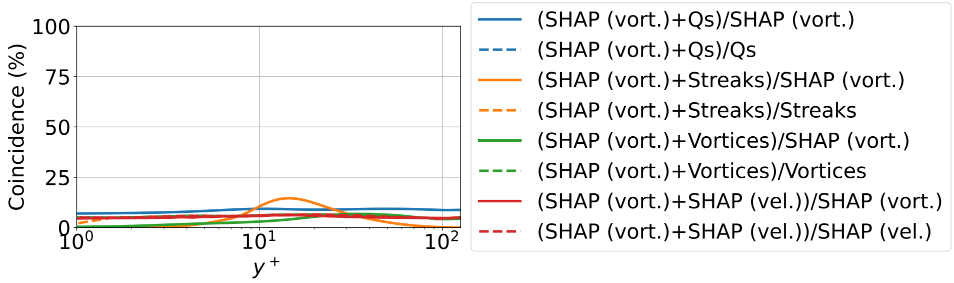

Supplementary Figure 21: **Coincidence of the coherent structures.** Percentage of coincidence of pairs of the following structures: SHAP for the vorticity, Q events, streaks, vortices and SHAP for the velocity, relative to the volume of each type of the pair for a turbulent channel at $\mathrm{Re}_\tau = 125$.

SHAP structures in the flow around a square wall-mounted obstacle

In the present study, the SHAP structures have been calculated for a turbulent channel flow at two different friction Reynolds numbers: $\mathrm{Re}_\tau = 125$ and $\mathrm{Re}_\tau = 550$. However, in order to illustrate the adaptability of the present methodology to any type of flow, the SHAP structures in the flow around a square wall-mounted obstacle are also analyzed. The analyzed database, described in detail in Refs. [10, 11], was obtained by means of a direct numerical simulation (DNS) at a Reynolds number $\mathrm{Re}_h = u_0 h/\nu = 2000$, where h is the height of the obstacle, u_0 the freestream velocity and ν the kinematic viscosity. We analyze a region of the domain on the leeward side of after the obstacle with size $2.86h \times 2h \times 1.24h$. The obstacle has a cross-section $0.25h \times 0.25h$. The methodology of the main paper is reproduced for the obstacle flow, detecting the various coherent structures and evaluating their coincidence. It can be observed that, up to the obstacle height (for $y < h$), the agreement between SHAP structures and Q events is slightly lower than that at $Re_\tau = 125$ (with a moderate increase in the shear layers at $y > h$). Regarding the streaks, the coincidence is much lower than in the case of the channel, since the main dynamics are within the wake, with the wall having a less important role. Finally, the vortices exhibit a low level of agreement with

the SHAP structures as in the channel case. This example highlights that the SHAP framework can identify the most important features in different flows, regardless of the roles played by the classical structures.

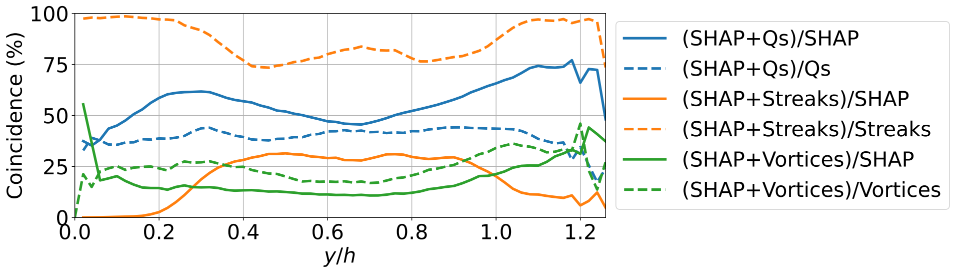

Supplementary Figure 22: **Coincidence of the various coherent structures in the flow around a square wall-mounted obstacle** [10, 11]**.** Percentage of coincidence of pairs of the following structures: SHAP, Q events, streaks and vortices, relative to the volume of each type of the pair.

Data availability

The minimum representative downsampled data used in this study will be made available open access at: https://github.com/KTH-FlowAI/XAI_turbulentchannel_3d_simplified.git. For the complete database, please contact the authors.

Code availability

The codes used for this work is available open access at: https://github.com/KTH-FlowAI/XAI_turbulentchannel_3d_simplified.git.

References

[1] Martínez-Sánchez, Á., Arranz, G., Lozano-Durán, A.: Decomposing causality into its synergistic, unique, and redundant components. Nature Communications **15**(1), 9296 (2024)

[2] Erion, G., Janizek, J.D., Sturmfels, P., Lundberg, S.M., Lee, S.-I.: Improving performance of deep learning models with axiomatic attribution priors and expected gradients. Nature machine intelligence **3**(7), 620–631 (2021)

[3] Lundberg, S.M., Lee, S.-I.: A unified approach to interpreting model predictions. Advances in neural information processing systems **30** (2017)

[4] Rashed-Al-Mahfuz, M., Moni, M.A., Uddin, S., Alyami, S.A., Summers, M.A., Eapen, V.: A deep convolutional neural network method to detect seizures and

characteristic frequencies using epileptic electroencephalogram (EEG) data. IEEE journal of translational engineering in health and medicine **9**, 1–12 (2021)

[5] Zheng, Q., Wang, Z., Zhou, J., Lu, J.: Shap-CAM: Visual explanations for convolutional neural networks based on Shapley value. In: European Conference on Computer Vision, pp. 459–474 (2022). Springer

[6] Lozano-Durán, A., Flores, O., Jiménez, J.: The three-dimensional structure of momentum transfer in turbulent channels. Journal of Fluid Mechanics **694**, 100–130 (2012)

[7] Kline, S.J., Reynolds, W.C., Schraub, F.A., Runstadler, P.W.: The structure of turbulent boundary layers. Journal of Fluid Mechanics **30**(4), 741–773 (1967) https://doi.org/10.1017/S0022112067001740

[8] Chong, M.S., Perry, A.E., Cantwell, B.J.: A general classification of three-dimensional flow fields. Journal of Physics A. **2**(5), 765–777 (1990)

[9] Osawa, K., Jiménez, J.: Causal features in turbulent channel flow. arXiv preprint arXiv:2405.15674 (2024)

[10] Martínez-Sánchez, Á., López, E., Le Clainche, S., Lozano-Durán, A., Srivastava, A., Vinuesa, R.: Causality analysis of large-scale structures in the flow around a wall-mounted square cylinder. Journal of Fluid Mechanics **967**, 1 (2023)

[11] Yousif, M.Z., Yu, L., Hoyas, S., Vinuesa, R., Lim, H.: A deep-learning approach for reconstructing 3d turbulent flows from 2d observation data. Scientific Reports **13**(1), 2529 (2023)

Acknowledgments

The authors acknowledge Adrián Lozano-Durán and Álvaro Martínez-Sánchez for their support with the validation of the causal nature of the SHAP values. The deep-learning-model training was enabled by resources provided by the National Academic Infrastructure for Supercomputing in Sweden (NAISS) at Berzelius (NSC), partially funded by the Swedish Research Council through grant agreement no. 2022-06725. The data has been obtained with support of grant PID2021-128676OB-I00 funded by MCIN/AEI/10.13039/ 501100011033 and by "ERDF A way of making Europe", by the European Union (SH). RV acknowledges the financial support from ERC grant no. 2021-CoG-101043998, DEEPCONTROL. Views and opinions expressed are however those of the author(s) only and do not necessarily reflect those of the European Union or the European Research Council. Neither the European Union nor the granting authority can be held responsible for them.

Author Contributions

Cremades, A.: Conceptualization, Methodology, Software, Validation, Investigation, Writing - Original Draft, Visualization. Hoyas, S.: Conceptualization, Data curation, resources, Writing - Original Draft, Funding acquisition. Vinuesa, R.: Initial idea, conceptualization, project definition, methodology, resources, Writing - Original Draft, Supervision, Project administration, Funding acquisition.

Competing Interests

The authors declare no competing interests.
